# Understanding atrioventricular septal defect: Anatomoechocardiographic correlation

**DOI:** 10.1186/1476-7120-6-33

**Published:** 2008-06-24

**Authors:** Nilda Espinola-Zavaleta, Luís Muñoz-Castellanos, Magdalena Kuri-Nivón, Candace Keirns

**Affiliations:** 1 Echocardiography in Out Patients Clinic, Instituto Nacional de Cardiología Ignacio Chávez, Juan Badiano N°1, Colonia Sección XVI, Mexico City, Mexico; 2 Embryology Department, Instituto Nacional de Cardiología Ignacio Chávez, Juan Badiano N°1, Colonia Sección XVI, Mexico City, Mexico; 3 Morphology Department, Escuela Superior de Medicina – IPN, Díaz-Mirón y Plan de San Luís, Colonia Casco de Santo Tomás, Tacuba, Mexico City, Mexico

## Abstract

**Objective:**

Correlate the anatomic features of atrioventricular septal defect with echocardiographic images.

**Materials and methods:**

Sixty specimen hearts were studied by sequential segmental analysis. Echocardiograms were performed on 34 patients. Specimen hearts with findings equivalent to those of echocardiographic images were selected in order to establish an anatomo-echocardiographic correlation.

**Results:**

Thirty-three specimen hearts were in situs solitus, 19 showed dextroisomerism, 6 were in situs inversus and 2 levoisomerism. Fifty-eight had a common atrioventricular valve and 2 had two atrioventricular valves. Rastelli types were determined in 21 hearts. Nine were type A, 2 intermediate between A and B, 1 mixed between A and B, 4 type B and 5 type C. Associated anomalies included pulmonary stenosis, pulmonary atresia atrial septal defect, patent ductus arteriosus and anomalous connection of pulmonary veins. Echocardiograms revealed dextroisomerism in 12 patients, situs solitus in 11, levoisomerism in 7 and situs inversus in 4. Thirty-one patients had common atrioventricular valves and three two atrioventricular valves. Rastelli types were established in all cases with common atrioventricular valves; 17 had type A canal defects, 10 type B, 3 intermediate between A and B, 1 mixed between A and B and 3 type C. Associated anomalies included regurgitation of the atrioventricular valve, pulmonary stenosis, anomalous connection of pulmonary veins, pulmonary hypertension and pulmonary atresia.

**Conclusion:**

Anatomo-echocardiographic correlation demonstrated a high degree of diagnostic precision with echocardiography.

## Background

Atrioventricular septal defect is characterized by a lack of separation between the right atrium and the left ventricle, creating communication between the two sides of the heart with a variety of aberrations of the atrioventricular valves, ventricular geometry, the fibrous skeleton and the cardiac conduction system [1]. This complex congenital heart defect can include atrial, atrioventricular and ventricular shunts, and rarely isolated ventricular shunts [2,3]. There are two basic forms of this endocardial cushion defect: a single, common AV valve and 2 valves within a common fibrous valve ring [4]. Both types present the same pathological features [1]. In some the atrioventricular septal defect is the confluence of three defects: ostium primum, atrioventricular septal defect and perimembranous and inlet ventricular septal defect, although the ostium primum defect may be absent [2,3]. When there are 2 atrioventricular valves the shunt occurs exclusively at the atrial level, such that the defect has traditionally been considered to be an ostium primum. However, these hearts have been shown to have structural deficiencies of the atrioventricular septa more than of the atrial septa. This concept is bolstered by the existence of anatomic specimens with this heart defect without ostium primum defects [1,3,5,6].

Echocardiography has provided precise images of the anatomic findings of this congenital malformation with details of the atrioventricular septal defect, the common atrioventricular valve or two separate atrioventricular valves, the relationships between the leaflets of the valves in both forms, septal structures and their defects, disproportion between the left ventricular inflow and outflow tracts, unwedged aorta and the shunts that determine the clinical presentation [2,5,6]. This anatomic information is of enormous value for the clinician in planning surgical treatment.

This study presents an anatomo-echocardiographic correlation that compares the anatomic features of specimen hearts with atrioventricular septal defect to echocardiographic images of equivalent findings in patients with this congenital malformation. These comparisons should aid in diagnostic interpretation [7-12].

## Materials and methods

Sixty specimen hearts from the Department of Embryology of the Instituto Nacional de Cardiología "Ignacio Chávez" with atrioventricular septal defect were examined. Each specimen was described following the guidelines of the segmental sequential system utilized in the diagnosis of congenital heart defects [13]. Atrial situs, atrioventricular and ventriculo-arterial connections, alterations of atrioventricular and arterial valves, subvalvular apparatus and morphologic characteristics of the ventricles, including atrial and ventricular septa, were analyzed.

Between May 2002 and December 2006 thirty-four adult patients from adult outpatient clinic with clinical suspicion of atrioventricular septal defect were studied. Fourteen were women and 20 men with an average age of 24.95 ± 6.94 years.

All patients underwent complete clinical history and transthoracic and/or transesophageal echocardiograms. The echocardiographic study was performed using a Philips Sonos 5500 machine with an S3 electronic probe and multiplane transesophageal probe.

Abdominal situs was determined from the subcostal plane, and the atrial situs on the basis of the morphology of the atrial appendages visualized in the apical 4-chamber view. Atrioventricular valve features and Rastelli type [14] were evaluated from the 4-chamber apical image. The characteristics of atrial and ventricular septal defects were assessed in 4 chamber apical and subcostal planes. Shunt types and the valvular regurgitation were evaluated with color Doppler. Ventriculo-arterial connections were studied in parasternal short axis at the level of great vessels and in the 5-chamber apical view. Stenosis and/or regurgitation of semilunar valves were evaluated with color and continuous wave Doppler in the apical 5-chamber view, and the severity of these lesions was classified according to the guidelines previously described [15].

When the patient had a poor acoustic window and/or when diagnosis was in doubt transesophageal echocardiography was performed using a multiplane probe.

Anatomo-echocardiographic correlation was demonstrated by establishing the correspondence between the anatomic feature of the specimen and the equivalent echocardiographic image from a patient with the same type of malformation.

## Results

### Morphological findings

Atrial situs was solitus in 33 hearts, 19 had dextroisomerism, 6 had situs inversus and 2 had levoisomerism. All presented a common fibrous atrioventricular ring. Fifty-eight hearts had a common atrioventricular valve, and two had two separate valves. All had atrioventricular septal defects. Hearts with common atrioventricular valves had ventricular septal defects. In those with two atrioventricular valves this ventricular septal defect was obliterated by the insertion of the left atrioventricular septal anterior and posterior leaflets in the crest of the ventricular septum. Ostium primum was present in 58 hearts. In two the atrial septum was well developed and there was no ostium primum. The two types of atrioventricular septal defect manifested all hallmarks of the atrioventricular septal defect: excavation of the ventricular septum, disproportion of the left ventricular inflow tract and outflow tract, unwedged aorta, a common fibrous ring, absence of the membranous septum, atrioventricular valves on the same plane, and elongation of the left ventricular outflow tract. In two specimens's insertion of the chordae tendineae of the left antero-superior leaflet caused obstruction of the left ventricular outflow tract.

Hearts with one valve had a five leaflet pattern: two (right and left) antero-superior leaflets, two (right and left) lateral leaflets and a common posterior leaflet. Hearts with two atrioventricular valves had a pattern of three leaflets.

Rastelli types were established in twenty-one hearts. Nine were type A, two were intermediate between A and B, one was a mixture of types A and B, four were type B and five were type C. Rastelli types were not determined in thirty-nine hearts because 10 had double inlet to a single ventricle, nine a right ventricular double inlet and eight a left ventricular double inlet. Eleven were surgically altered and one had deterioration.

Atrioventricular and ventriculo-arterial connections as well as associated anomalies are shown in Table 1.

**Table 1:** Morphological characteristics of atrioventricular septal defects

**n = 60**	**Anatomic finding**	**n**	**%**
Position (Situs)	Solitus	33	55
	Inversus	6	10
	Dextroisomerism	19	32
	Levoisomerism	2	3
AV Connection	Concordant	27	45
	SVDI	10	17
	RVDI	9	15
	LVDI	8	14
	Ambiguous	5	8
V-A Connection	RVDO	21	35
	Concordant	19	32
	SVDO	11	18
	Discordant	9	15
Associated anomalies	Pulmonary stenosis	19	32
	Pulmonary atresia	10	17
	Patent foramen ovale	9	15
	Patent ductus arteriosus	5	8
	Anomalous connection of pulmonary veins	5	8
	AV Valve dysplasia	1	1.6
	Obstruction of the LV outflow tract	1	1.6

A-V: Atrioventricular; SVDI: Single ventricular double inlet; RVDI: Right ventricular double inlet; LVDI: Left ventricular double inlet; V-A: Ventriculo-arterial; RVDO: Right ventricular double outlet; SVDO: Single ventricular double outlet.

### Echocardiographic findings

Echocardiographic studies showed dextroisomerism in twelve patients, atrial situs solitus in eleven, levoisomerism in seven and situs inversus in four. Thirty-one patients had common atrioventricular valves and three had two separate valves.

All of the patients had atrioventricular septal defects. Ventricular septal defects were found in the group with common atrioventricular valve; obliterated in the group with two atrioventricular valves by the insertion of the septal anterior and posterior leaflets of the left atrioventricular valve. Ostium primum was present in thirty-one hearts, while in three the atrial septum was well developed and no ostium primum was found.

As in the anatomic specimen hearts, patients with a common atrioventricular valve had a five leaflet pattern: two (right and left) antero-superior leaflets, two (right and left) lateral leaflets and a common posterior leaflet. Hearts with two atrioventricular valves had a pattern of three leaflets.

Rastelli types were determined in all cases with common atrioventricular valves. Seventeen had type A, ten type B, three intermediate between A and B, three type C and one a mixture of types A and B.

Atrioventricular and ventriculo-arterial connections and associated anomalies are shown in Table 2.

**Table 2:** Echocardiographic characteristics of atrioventricular septal defects

**n = 34**	**Anatomic finding**	**n**	**%**
Position (Situs)	Solitus	11	32
	Inversus	4	12
	Dextroisomerism	12	35
	Levoisomerism	7	21
AV Connection	Concordant	15	44
	RVDI	8	23
	SVDI	4	12
	Ambiguous	4	12
	LVDI	3	9
V-A Connection	RVDO	14	41
	Concordant	10	29
	Discordant	6	18
	SVDO	4	12
Associated anomalies	Pulmonary stenosis	14	41
	Pulmonary atresia	5	15
	Pulmonary hypertension	7	20
	Patent ductus arteriosus	2	6
	Anomalous connection of pulmonary veins	9	26
	AV valve dysplasia	2	6
	Valve regurgitation	15	44
	Obstruction of the LV outflow tract	1	3
	Single coronary ostium	1	3

A-V: Atrioventricular; SVDI: Single ventricular double inlet; RVDI: Right ventricular double inlet; LVDI: Left ventricular double inlet; V-A: Ventriculo-arterial; RVDO: Right ventricular double outlet; SVDO: Single ventricular double outlet.

### Anatomo-echocardiographic correlation

A comparison of the anatomic features of the atrioventricular canal with corresponding images obtained from patients with the same condition showed strong correlation.

**Figure 1:**
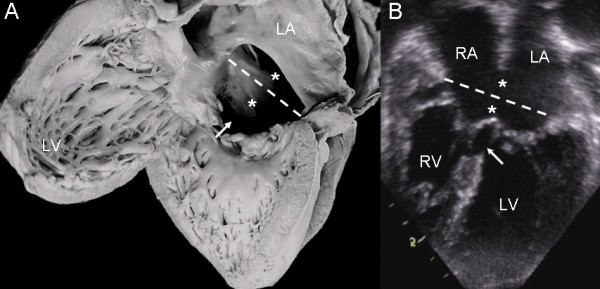
**Atrioventricular septal defect with common atrioventricular valve, Rastelli type A**. (A): Internal view of left heart chambers. The dotted line indicates the plane of the atrioventricular junction. The upper asterisk corresponds to the ostium primum, the lower asterisk to the atrioventricular septal defect and the arrow to the ventricular septal defect. (B): Four chamber echocardiographic image in situs solitus shows Rastelli type A insertion. Observe the same features as in the anatomic specimen. Abbreviations: LA: Left atrium; RA Right atrium; LV: Left ventricle; RV: Right ventricle.

In the common valve type the chambers open into a wide canal with ostium primum, atrioventricular septal defect and ventricular septal defect. In Rastelli type A the chordae tendineae of the anterior leaflets insert in the crest of the ventricular septum (Fig. 1A). The apical 4-chamber echocardiographic image shows the confluent septal defects, common atrioventricular junction with insertion of the anterior leaflets in the crest of the septum (Rastelli type A) and ventricular septal defect in the interchordal spaces (Fig. 1B).

**Figure 2:**
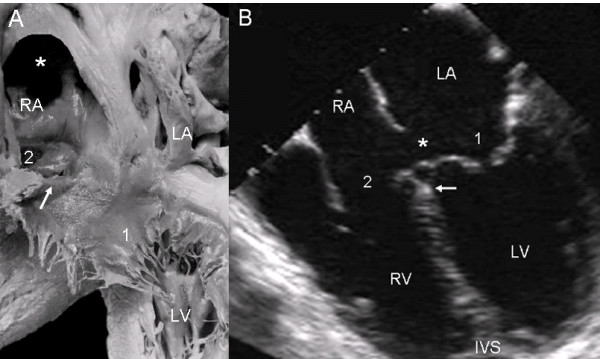
**Atrioventricular septal defect with two separate atrioventricular valves**. (A): Internal view of left chambers, left (1) and right (2) atrioventricular valves, ostium primum (asterisk), and the arrow pointing to the insertion of the anterior septal leaflet in the crest of the interventricular septum. (B): The 4-chamber transesophageal image shows the same characteristics as in the anatomic specimen. IVS: Interventricular septum. Other abbreviations as before.

In the atrioventricular septal defect with two separate valve orifices these are contained within the common atrioventricular junction. The wide septal defect includes the ostium primum and the atrioventricular septal defect. The septal leaflets of the left atrioventricular valve insert in a continuous manner in the crest of the ventricular septum, obliterating the ventricular septal defect (Fig. 2A). The apical 4-chamber echocardiographic image shows the two separate valve orifices, the fusion of the septal leaflets on the crest of the ventricular septum and the wide defect with two confluent components: the ostium primum and the atrioventricular septal defect (Fig. 2B).

**Figure 3:**
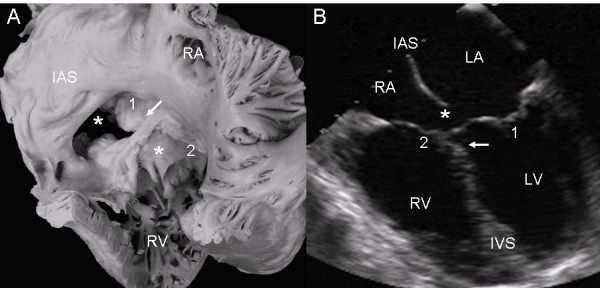
**Atrioventricular septal defect with two separate atrioventricular valves**. (A): Internal view of right chambers, left (1) and right (2) atrioventricular valves, ostium primum (asterisk) and arrow pointing to the insertion of the septal leaflets of the left valve in the crest of the interventricular septum. Note the confluence of the ostium primum with the atrioventricular septal defect (upper asterisk), the commissure that separates those leaflets, the ample commissure between the septal leaflets and the anterior leaflet of the right atrioventricular valve (lower asterisk). (B): The two separate atrioventricular valves (1 and 2) and the fusion of the septal leaflets to the crest of the interventricular septum that closes the ventricular septal defect (arrow) can be seen. IAS: Interatrial septum. Other abbreviations as before.

The view of the atrioventricular septal defect with two valve orifices from the right chambers shows a well-developed atrial septum. The fusion of the septal leaflets of the left atrioventricular valve on the crest of the ventricular septum and the commissure that separates these septal leaflets, as well as the broad commissure between the septal and anterior leaflets of the right atrioventricular valve can be seen through the wide septal defect (Fig. 3A). The 4-chamber echocardiographic image shows the intact atrial septum and the atrioventricular septal defect without the ostium primum (Fig. 3B).

**Figure 4:**
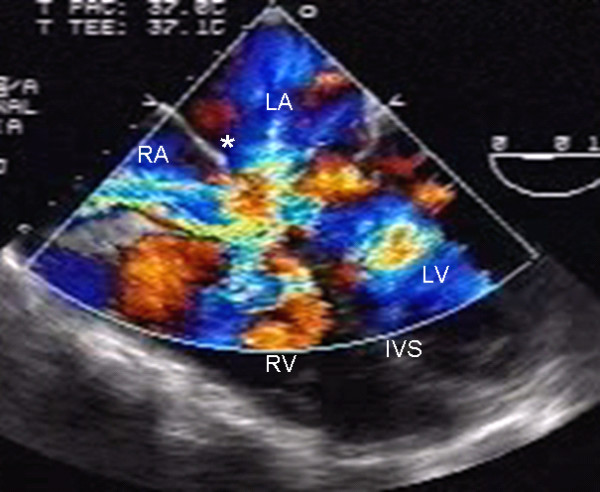
Two-dimensional transesophageal echocardiogram at 0° with color Doppler shows the shunt above the valve plane. Note the interatrial septum (asterisk). Abbreviations as before.

The two-dimensional echocardiographic apical 4-chamber image with color Doppler of an atrioventricular septal defect with two valve orifices makes it possible to observe the atrial shunt above the atrioventricular valves (Fig. 4).

**Figure 5:**
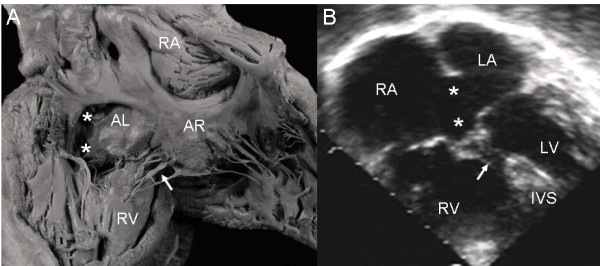
**Atrioventricular septal defect with a Rastelli type A common atrioventricular valve**. (A) Internal view of right heart chambers. Note the small ostium primum (upper asterisk) continuous with atrioventricular septal defect (lower asterisk) and interchordal spaces (arrow). (B) The 4-chamber echocardiographic image shows the same characteristics seen in the anatomic specimen. Abbreviations as before.

In the atrioventricular canal with Rastelli type A common valve the chordae tendineae of the left and right anterior leaflets insert on the crest of the ventricular septum and the interchordal spaces form the ventricular septal defect. The broad ventricular septal defect is continuous with the ostium primum and the atrioventricular septal defect (Fig. 5A). The same features seen in the specimen heart appear in the 4-chamber echocardiographic image (Fig 5B).

**Figure 6:**
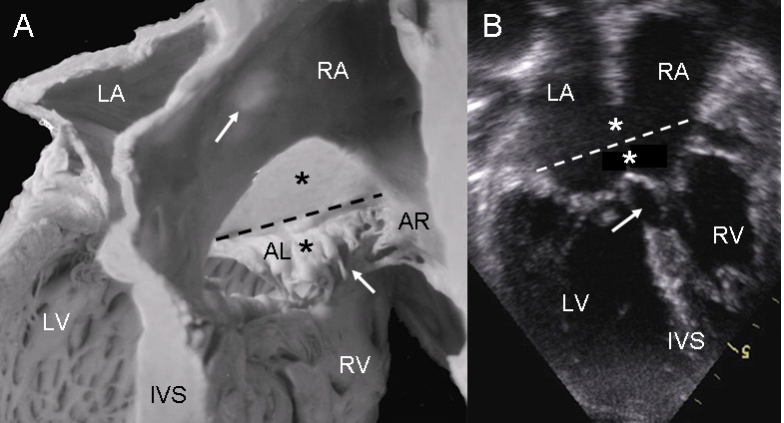
**Atrioventricular septal defect with a common atrioventricular valve with Rastelli type A insertion**. (A). The dotted line indicates the common atrioventricular junction. Above it are the ostium primum (upper asterisk), the atrioventricular septal defect itself (lower asterisk) and the ventricular septal defect in the interchordal spaces (lower arrow). The upper arrow points to the region of the fossa ovale. (B) The 4-chamber transthoracic image shows the same features as in the anatomic specimen. Abbreviations: AL: Anterior left; AR: Anterior right. Other abbreviations as before.

The anatomic features of a Rastelli type A atrioventricular septal defect had a precise correlation with the echocardiographic image (Fig. 6).

**Figure 7:**
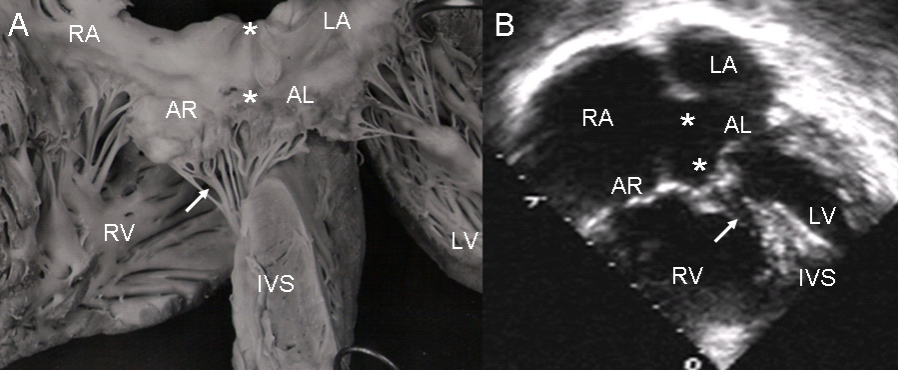
**Atrioventricular septal defect with a common valve and intermediate insertion between Rastelli types A and B**. (A) The insertion of the anterior leaflets is on the right surface of the ventricular septum below its crest. The left anterior leaflet bridges the ventricular septal defect. The ostium primum (upper asterisk) and the atrioventricular defect (lower asterisk) combine to form the large septal defect. The arrow points to the interchordal spaces. (B) The 4 chamber echocardiographic image shows an insertion similar to that observed in the anatomic specimen (arrow). Abbreviations as before.

In an atrioventricular septal defect with common valve the insertion of the anterior leaflet chordae tendineae on the right side of the ventricular septum near the crest represent an intermediate insertion between Rastelli types A and B (Fig. 7A). The correspondence with the echocardiographic image in situs solitus can be appreciated (Fig. 7B).

**Figure 8:**
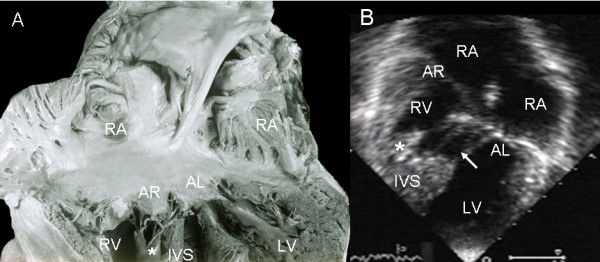
**Atrioventricular septal defect with common atrioventricular valve in dextroisomerism**. (A) Note the mixed insertion of the anterior leaflets in the crest of the ventricular septum (Rastelli type A) and a papillary muscle (asterisk) adjacent to the ventricular septum (Rastelli type B). (B) Echocardiographic 4-chamber image in dextroisomerism shows a similar insertion.

When the atrioventricular septal defect with common valve is in dextroisomerism the insertion of the chordae tendineae occurs at two sites, in the crest of the ventricular septum (Rastelli type A) and in the papillary muscle adjacent to the ventricular septum (Rastelli type B, Fig. 8A). The 4-chamber echocardiographic images show the same type of insertion as the anatomic specimen (Fig. 8B).

**Figure 9:**
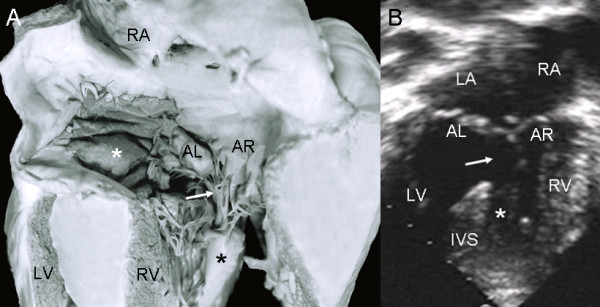
**Atrioventricular septal defect with common valve with Rastelli type B insertion**. (A) View of right heart chambers. Surgical patch is visible over atrioventricular septal defect (white asterisk) and the insertion of anterior leaflets to a papillary muscle (black asterisk) joined to the apical portion of the ventricular septum. (B) The two-dimensional echocardiographic 4-chamber image shows a similar insertion. Abbreviations as before.

In a Rastelli type B atrioventricular septal defect, the view of the anatomic specimen from the right chambers shows a surgical patch and the insertion of the left anterior leaflet (bridging leaflet) to the papillary muscle tethered to the apical portion of the ventricular septum (Fig. 9A). In the 4-chamber echocardiographic image the left anterior leaflet bridges the ventricular septal defect and inserts in the papillary muscle (Fig 9B).

**Figure 10:**
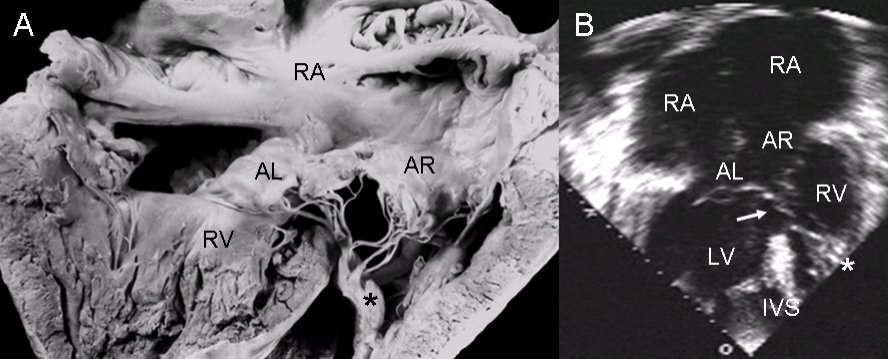
**Atrioventricular septal defect with common valve with Rastelli type C insertion**. (A) Right heart chambers with insertion of the anterior leaflets in a papillary muscle (asterisk) joined to the right ventricular free wall. (B) Echocardiographic view of four chambers in dextroisomerism shows a similar insertion. Abbreviations as before.

In a view from the right chambers of a Rastelli type C atrioventricular septal defect the left anterior leaflet can be seen to pass over the ventricular septum and insert in the papillary muscle in the right ventricular free wall (Fig. 10A). The 4-chamber echocardiographic image in dextroisomerism shows the left anterior leaflet as a bridge over the ventricular septum and its chordae tendineae inserting in the parietal papillary muscle (Fig. 10B).

**Figure 11:**
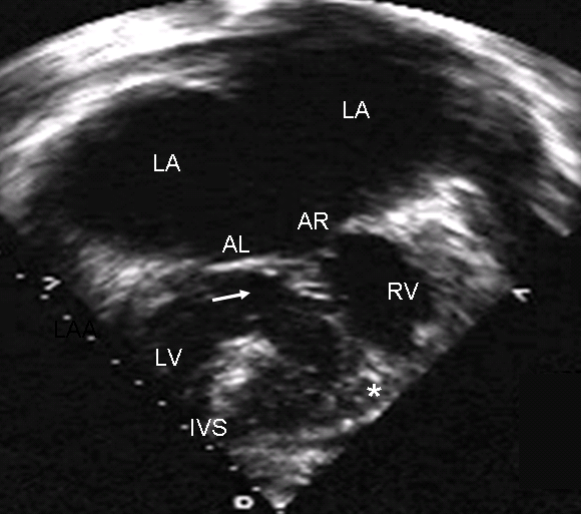
**Atrioventricular septal defect with common valve and Rastelli type C insertion in levoisomerism**. Two-dimensional 4-chamber image shows the insertion of the anterior leaflets to two papillary muscles (asterisk) joined to the right ventricular free wall. The arrow points to the ventricular septal defect. Abbreviations as before.

Figure 11 shows a heart with a Rastelli type C atrioventricular septal defect in levoisomerism.

**Figure 12:**
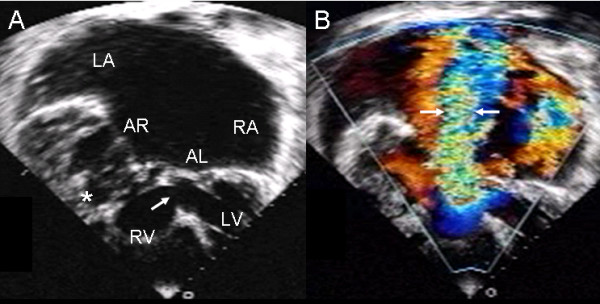
**Atrioventricular septal defect with Rastelli type C common valve in situs inversus**. (A) Two-dimensional 4 chamber image shows situs inversus and the insertion of the anterior leaflets to two papillary muscles (asterisk) joined to the right ventricular free wall. The arrow points to the ventricular septal defect beneath the leaflet bridge. (B) With color Doppler the severe regurgitation of the common atrioventricular valve can be seen (arrows) Abbreviations as before.

The Rastelli type C insertion of anterior leaflets and ventricular septal defect are shown in an atrioventricular septal defect with common atrioventricular valve in situs inversus (Fig. 12A). Color Doppler demonstrates the severe regurgitation of the common AV valve (Fig. 12B).

**Figure 13:**
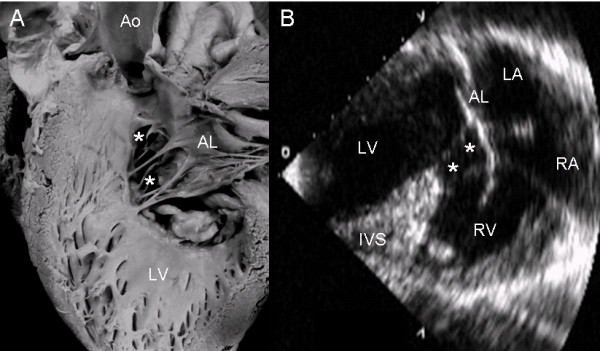
**Atrioventricular septal defect with Rastelli type A common valve**. (A) The left ventricle is visible with the excavated appearance of the interventricular septum and the interchordal spaces (asterisks) that create the ventricular septal defect. (B) The two-dimensional echocardiographic 4-chamber image shows the same features as in the anatomic specimen. Abbreviations as before.

In atrioventricular septal defect with common atrioventricular valve the "scooped out" appearance of the ventricular septum can be seen, as well as the interchordal spaces forming the ventricular septal defect (Fig. 13A). The 4-chamber echocardiographic image shows the same features (Fig. 13B).

**Figure 14:**
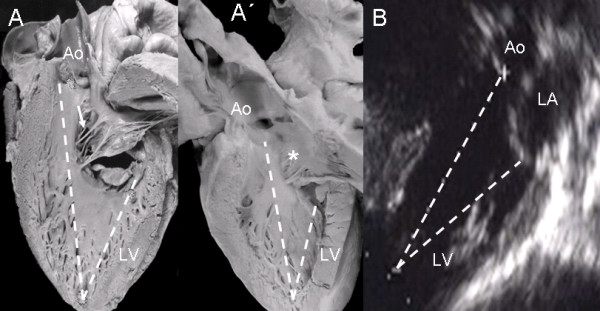
**Hearts anatomic specimens with a common atrioventricular valve (A) and with two atrioventricular valves (A'), in the latter observe a continuous valve insertion on the crest of the ventricular septum, obliterating the ventricular septal defect (asterisk)**. Note the similarity in both types of atrioventricular defect of the shortened inflow tract and the elongated outflow tract (dotted line). The same features are shown in the echocardiographic image (B) of a common atrioventricular valve. Abbreviations as before.

A comparison of the left ventricles in atrioventricular septal defect with common atrioventricular valve and with two separate valves shows the similarity of the insertions of left anterior leaflets in the two types. In the former there are interchordal spaces and in the latter continuous insertion and obliterated ventricular septal defect. In both types the atrioventricular septal defect shows a similarity in the shortening of the inlet and elongation of the outlet (Fig 14A). The echocardiographic image shows the same "goose neck" type malformation (Fig. 14B).

**Figure 15:**
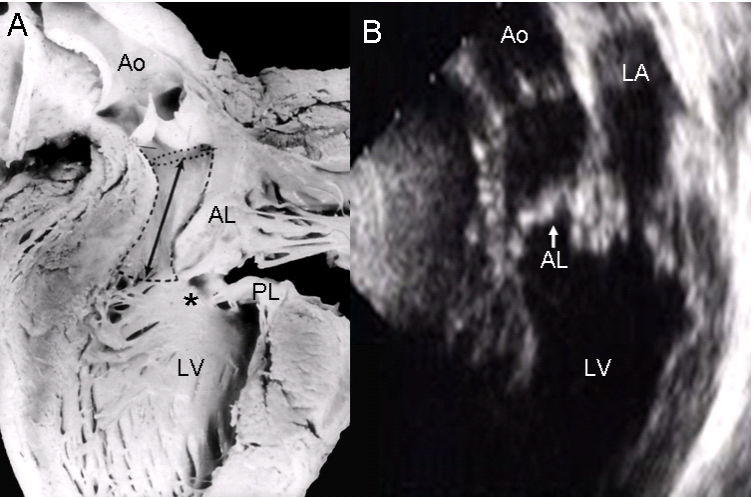
**Atrioventricular septal defect with two valve orifices.** (A) Internal view of the left ventricle showing obstruction of the outflow tract by invasion of the left anterior leaflet of the left atrioventricular valve (dotted lines and double headed arrow). The asterisk indicates the commissure between the septal anterior and posterior leaflets. (B) The parasternal long axis echocardiographic image shows similar features (arrow). Abbreviations: PL: Posterior leaflet. Other abbreviations as before.

Obstruction of the left ventricular outflow tract by encroachment of the left anterior leaflet of the left atrioventricular valve can be observed in an atrioventricular canal with two valve orifices (Fig. 15A). The parasternal long axis echocardiographic image shows the same kind of obstruction of the left ventricular outflow tract by the insertion of the left anterior leaflet (Fig. 15B).

**Figure 16:**
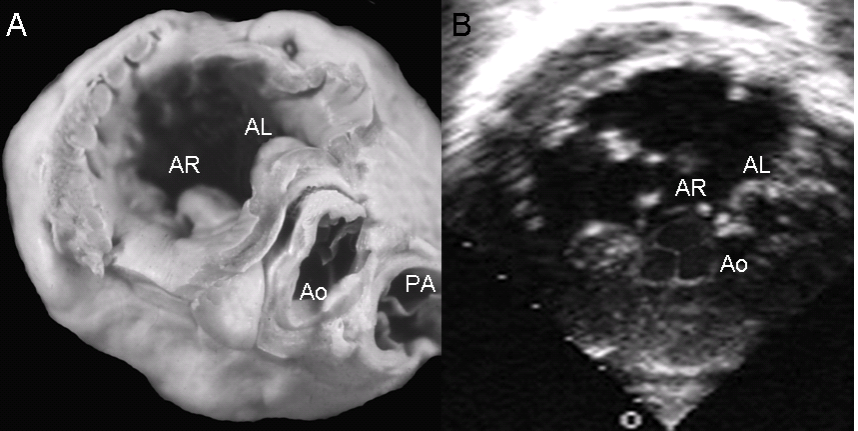
**Atrioventricular septal defect with common valve.** (A) Note the dilatation of the atrioventricular ring with unwedging of the aortic valve. (B) Parasternal short axis echocardiographic image with the same characteristics as in the specimen. Abbreviations: PA: Pulmonary artery. Other abbreviations as before.

In an atrioventricular septal defect with common atrioventricular valve dilatation of the valve ring produces unwedging of the aortic valve and the fibrous continuity between this valve and the left anterior leaflet of the common atrioventricular valve (Fig. 16A). The parasternal short axis echocardiographic image mirrors the findings of the specimen (Fig. 16B).

**Figure 17:**
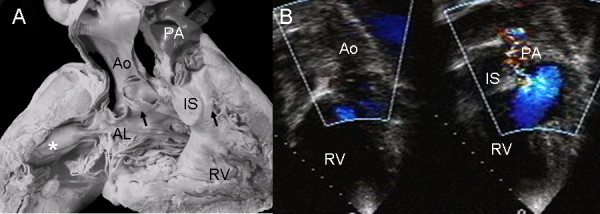
**Atrioventricular septal defect with common atrioventricular valve, double outlet right ventricle and Fallot's tetralogy.** (A) Anatomic specimen. (B) The echocardiographic image shows a similar features as in the anatomic specimen. Abbreviations: IS: Infundibular septum. Other abbreviations as before.

As this anatomic specimen shows, atrioventricular septal defect with common valve can be associated with right ventricular double outlet and Fallot's tetralogy. A Rastelli type C insertion of the left anterior leaflet, pulmonary stenosis and infundibular septum separating the two outflow tracts can be observed (Fig. 17A). The echocardiographic image shows the right ventricular double outlet type ventriculo-arterial connection with stenosis of the pulmonary valve (Fig. 17B).

**Figure 18:**
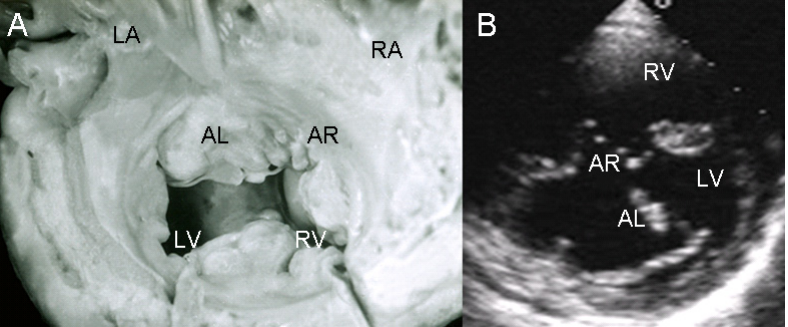
**Atrioventricular septal defect with a balanced form common valve**. (A) The common valve is equally shared by the two ventricles. (B) The echocardiographic image at the level of both ventricles shows the same features that are apparent in the anatomic specimen. Other abbreviations as before.

The atrioventricular septal defect can have a common atrioventricular valve that has equal connections to the two ventricles (Fig. 18A). The echocardiographic image at the level of the two ventricles demonstrates the same features as the anatomic specimen (Fig. 18B).

**Figure 19:**
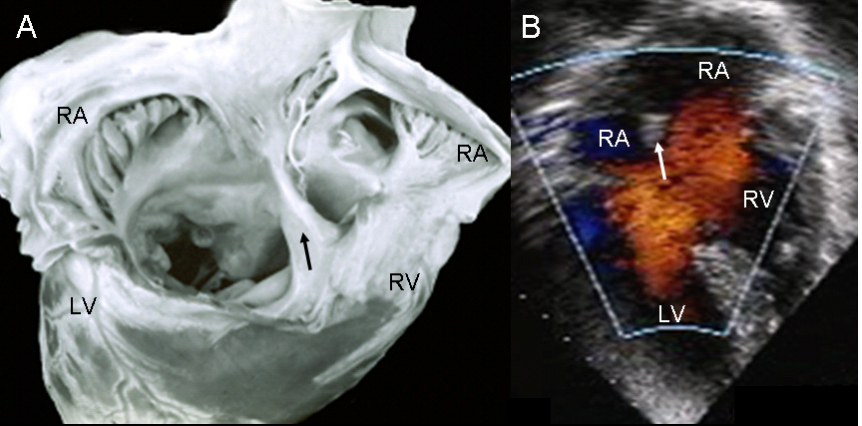
**Atrioventricular septal defect with a common atrioventricular valve in dextroisomerism with left dominance.** (A) In the postero-superior view of both atria with anatomically right characteristics, the valve ring is displaced toward the left ventricle, and there is an interatrial band typical of this atrial situs (arrow). (B) The two-dimensional 4-chamber echocardiographic image with color Doppler shows the same characteristics evident in the anatomic specimen with the shunt across the atrioventricular septal defect. Abbreviations as before.

Left dominance is evident in an atrioventricular septal defect with common atrioventricular valve in dextroisomerism. Note that in the postero-superior view both atria have anatomically right characteristics, the valve ring is displaced toward the left ventricle and there is an atrial septal band that is typical of this atrial situs (Fig. 19A). The two dimensional 4-chamber echocardiographic image with color Doppler shows the characteristics described in the anatomic specimen and the shunt through the atrioventricular septal defect (Fig. 19B).

**Figure 20:**
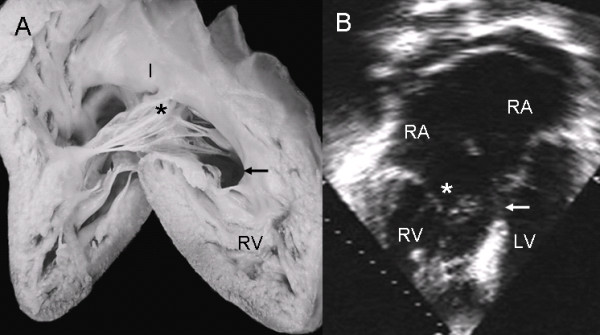
**Atrioventricular septal defect with a common atrioventricular valve in dextroisomerism with right dominance**. (A) Note the preponderance of the connection of the common atrioventricular valve with the right ventricle with a double inlet. (B) Four chamber echocardiographic image shows the same features observed in the anatomic specimen. Abbreviations as before.

An atrioventricular septal defect can also have right dominance in dextroisomerism. The internal view of the right ventricle reveals the predominant connection of the common atrioventricular valve with this chamber (Fig. 20A). The same right ventricular dominance with dextroisomerism is shown in the 4-chamber echocardiographic image (Fig. 20B).

## Discussion

The atrioventricular septal defect occurs as a consequence of detained growth and development of the postero-inferior and antero-superior mesenchymal endocardial cushions of the atrioventricular canal. Thus, their fusion, which in the normal heart constitutes the atrioventricular septum separating the right atrium from the left ventricle, does not occur. Instead, a large septal defect is established that includes components at the atrioventricular and ventricular levels and in many cases at the atrial level (ostium primum) [2,3].

**Figure 21:**
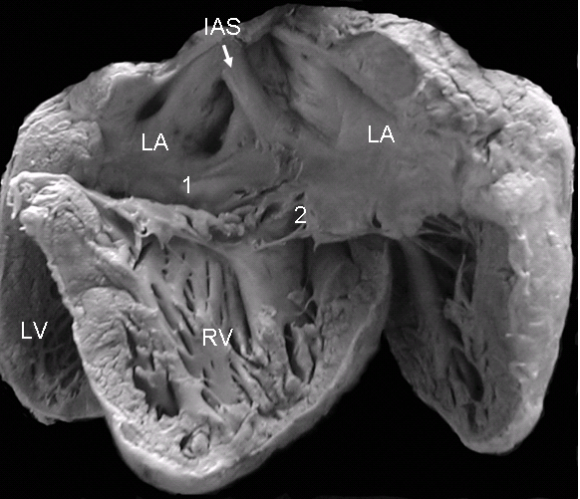
Anatomic specimen shows levoisomerism with left (1) and right (2) atrioventricular valves. Note the common atrium. Abbreviations as before.

This congenital heart defect can be found in any position (situs) of the heart: situs solitus, situs inversus or in the isomeric positions of dextroisomerism or levoisomerism (Fig. 11, 21). In all four positions it can occur in its two forms, either with a common atrioventricular valve or two separate atrioventricular valves within the common atrioventricular junction. All of these variations are easily demonstrated with echocardiography.

It is noteworthy that the pathological stigmata of the two anatomic forms of atrioventricular septal defect are practically identical. When the atrioventricular leaflets are removed from the atrioventricular ring in the basal portion of the ventricular mass there is no way to determine whether the patient to whom the heart pertained had a so called "partial" or "complete" type of atrioventricular canal [16]. This classification, as well as the classic denominations, has fallen into disuse [17,18].

It is the relationships among the leaflets within the common atrioventricular junction that determine the number of atrioventricular orifices, while the relationships between the leaflets and the septal structures dictate the possible shunts that can be established between the two sides of the heart [2,19-22].

Other anatomic characteristics that can be determined by ultrasound involve the septal structures and their defects. Classic cases manifest a lack of separation between the left ventricle and the right atrium, as the term atrioventricular septal defect suggests. The absence of the membranous septum and an incomplete muscular ventricular septum create a large perimembranous and inflow tract ventricular septal defect in cases of atrioventricular septal defect with common atrioventricular valve that is obliterated when there are two atrioventricular valves. Finally, failure of closure of the ostium primum originates a large atrial septal defect, although in some cases the ostium primum is absent and the septum is complete [3]. The condition of the atrial septum can be examined with a 4-chamber image at the level of the atrioventricular junction and the inferior portion of the atrial septum. If the septum is complete its inferior portion reaches the atrioventricular junction and there is no ostium primum. This condition can be seen in one of our echocardiographic images. When the atrial septum is deficient its inferior portion is far above the plane of the atrioventricular junction and an ostium primum is present. Cases have been reported in the literature in which more than an atrial septal defect there is a defect in the atrioventricular septum [2,19,20]. In those cases in which the atrial septum fused with the leaflets of the common atrioventricular valve, a shunt would only be possible through a ventricular septal defect. One case is mentioned in the literature in which the septal defect was obliterated by fusion of the atrioventricular valve leaflets to the cardiac septa, and the patient had no manifestations of this congenital malformation [22].

In cases of atrioventricular septal defect with a common atrioventricular valve and a ventricular septal defect the mode of insertion of the right and left anterior leaflets determines the Rastelli type, a classification with significance for surgical correction. In fact, Rastelli types A, B and C constitute the most frequent sites of insertion in a spectrum of possibilities. In this study intermediate insertions between types A and B and mixed A and B insertions in the same heart are shown.

As far as the dominance of the left (Fig. 19) or right (Fig. 21) ventricle is concerned, this should be considered in the setting of double inlet ventricle.

Other key characteristics of this cardiac anomaly that are present in types with one and two valve orifices are the unwedged aorta with respect to the atrioventricular valves with a "goose neck" deformity of the left ventricular out flow tract. This is caused by displacement of the common atrioventricular fibrous ring and elongation and displacement of the distance between the apex and the aortic valve. With the echocardiographic 5-chamber apical image it is easy to identify the decrease in the ventricular inlet that reflects a disproportion between it and the elongated outlet: the greater shortening of the inlet greater the elongation of the outlet. This swan's neck shape constitutes a potential postoperative risk of obstruction [2,23]. On occasion the chordae tendineae of the anterior leaflet insert in the interior of the outflow tract, as can be seen in two of our specimens and one of our patients.

The problems of clinical diagnosis can be solved by cross-sectional echocardiography that shows the classic anatomic features of the atrioventricular defect [2,19,24].

In conclusion, this group of lesions is unified by the anatomical hallmarks of a common atrioventricular junction and deficient atrioventricular septation. An understanding of the anatomy of this congenital malformation is mandatory for clinical diagnosis by imaging. The anatomo-echocardiographic correlation between specimen hearts and echocardiograms of patients with equivalent findings demonstrates the high degree of precision that can be achieved with echocardiographic diagnosis. Validation by anatomic specimens is the defining reference for the imaging that is indispensable for diagnosis, treatment and follow-up of these patients.

## Authors' contributions

LMC and NEZ participated to the written manuscript, MKN made the photographs of the heart specimens and helped to draft the manuscript and CK helped to draft the manuscript and made the translation from Spanish into English. All the authors read and approved the final manuscript.
